# Lipids and organic acids in three gut locations affect feed efficiency of commercial pigs as revealed by LC–MS-based metabolomics

**DOI:** 10.1038/s41598-021-87322-8

**Published:** 2021-04-08

**Authors:** Yong Ye, Jie Wu, Jianping Quan, Rongrong Ding, Ming Yang, Xingwang Wang, Shenping Zhou, Zhanwei Zhuang, Sixiu Huang, Ting Gu, Lingjun Hong, Enqin Zheng, Zhenfang Wu, Jie Yang

**Affiliations:** 1 College of Animal Science and National Engineering Research Center for Breeding Swine Industry, South China Agricultural University, Guangdong, 510642 China; 2 Lingnan Guangdong Laboratory of Modern Agriculture, Guangzhou, 510642 China; 3 Guangdong Wens Breeding Swine Technology Co., Ltd., Guangdong, 527400 China

**Keywords:** Bioinformatics, Metabolomics

## Abstract

Feed efficiency (FE) is an important economic indicator in pig production. Improving the FE of commercial pigs is an important strategy for minimizing pig production costs and providing sustainability to the pig industry. In this study, nontargeted LC–MS metabolomics was performed on the contents of the three intestine segments (ileum, cecum and colon) of high-FE and low-FE pigs to explore the effects of small-molecule metabolites in pig intestine on pig FE. A total of 225 Duroc × (Landrace × Yorkshire) pigs in the 30–100 kg stage were sorted based on FE, and 20 pigs with extreme phenotypes were selected, with 10 in each group. A total of 749 metabolites were identified, of which 15, 38 and 11 differed between high-FE and low-FE pigs in ileum, cecum and colon, respectively. These candidate biomarkers mainly comprised lipids and organic acids, which could partially explain the FE difference between the two groups. Among the identified differential metabolites, the lipids are mainly involved in combatting inflammation and oxidation in the ileum and cecum and in bile acid metabolism and vitamin D absorption in the cecum. A difference in organic acids was mainly observed in the hindgut, which is involved in the metabolism of amino acids and fatty acids. This comprehensive study provides new insight into the biochemical mechanisms associated with pig FE.

## Introduction

Feed costs are a major component of pig production costs^1^. Feed efficiency (FE), an important economic trait in pig production, is often indirectly measured as either the feed conversion ratio (FCR) or the residual feed intake (RFI)^2,3^. Low FE can cause the excessive release of nutrients, such as phosphorus and nitrogen, into the environment, causing environmental pollution^4^. In addition, low-FE pigs consume more feed than high-FE individuals before reaching the market weight standard and thus reduce the income and efficiency of pig producers. Therefore, improving FE is an essential way to reduce pig production costs and provide sustainability to the pig industry.

The FE of pigs is affected by many factors, including pig genetics, disease, production management, and intestinal microorganisms^5–7^. In addition, gut microbiota affect host metabolism and health through the production of metabolites^8^. Our previous studies have shown that the microbiota composition and functions in different intestinal locations of Duroc × (Landrace × Yorkshire) (DLY) pigs affect intestinal physiological function, and many microbes were identified that can potentially affect pig FE^9^. In this study, we further investigated the correlation between the intestinal contents of metabolites and FE in different intestinal regions through nontargeted metabolome detection.

The microbiota, microbial metabolites, secretions of the host gastrointestinal tract, and exogenous nutritional molecules constitute a complex and dynamic intestinal environment^10^. Untargeted and targeted mass spectrometry-based metabolomics enables the analysis of large numbers of small-molecule metabolites in biological samples. Compared with nuclear magnetic resonance (NMR), mass spectrometry (MS) is more sensitive and can measure higher numbers of molecules in samples^11^. Gas chromatography-mass spectrometry (GC–MS) can obtain better separation of metabolites than the liquid chromatography-mass spectrometry (LC–MS) when the derivatization information of metabolic species is provided before chemical analysis. In general, no derivatization is performed in LC–MS metabolic profiling, but it can be applied for more targeted analysis or to increase selectivity or sensitivity^12^. In this study, we used nontargeted LC–MS to identify the composition of metabolites in different intestinal locations and explore the metabolites potentially associated with pig FE.

## Result

### Phenotypic variables

The twenty selected pigs were evaluated for feeding efficiency and growth traits. There were significant differences in AFI, FCR and RFI between the high-FE group and the low-FE group (*P* < 0.05). The average AMBW and ADG of the HFE group were higher than those of the LFE group, but the differences were not significant (*P* = 0.12 and 0.17, respectively) (Table 1).

**Table 1:** Descriptive statistics of high feed efficiency (HFE) and low feed efficiency (LFE) for phenotypic traits.

Trait	Unit	HFE(± sd)	LFE(± sd)	*P *value
ADG †	kg/day	0.84(± 0.05)	0.81(± 0.10)	0.17
AMBW‡	kg	23.00(± 0.19)	22.86(± 0.16)	0.12
DFI‡	kg/day	1.83(± 0.12)	2.03(± 0.13)	0.004*
RFI‡	kg	− 0.16(± 0.06)	0.07(± 0.15)	0.001*
FCR†	kg/kg	2.23(± 0.07)	2.64(± 0.06)	1.08E−5*

*ADG* average daily gain, *AMBW* average metabolic body weight gain, *DFI* daily feed intake, *RFI* residual feed intake, *FCR* feed conversion ratio.

*P < 0.05.

^†^Student's t-test.

^‡^Wilcoxon Test.

### Metabolome profile

**Figure 1:**
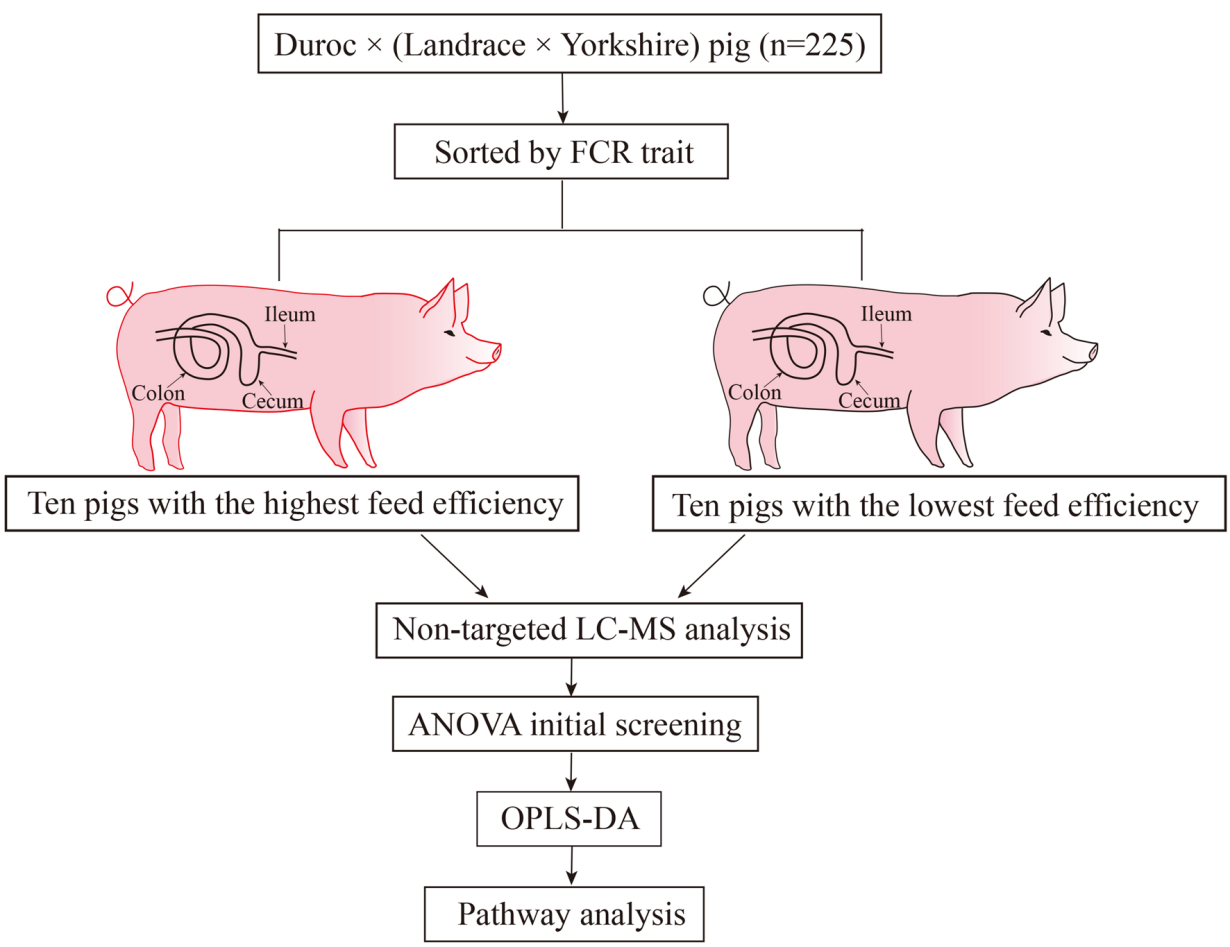
Overview of the experimental design.

**Figure 2:**
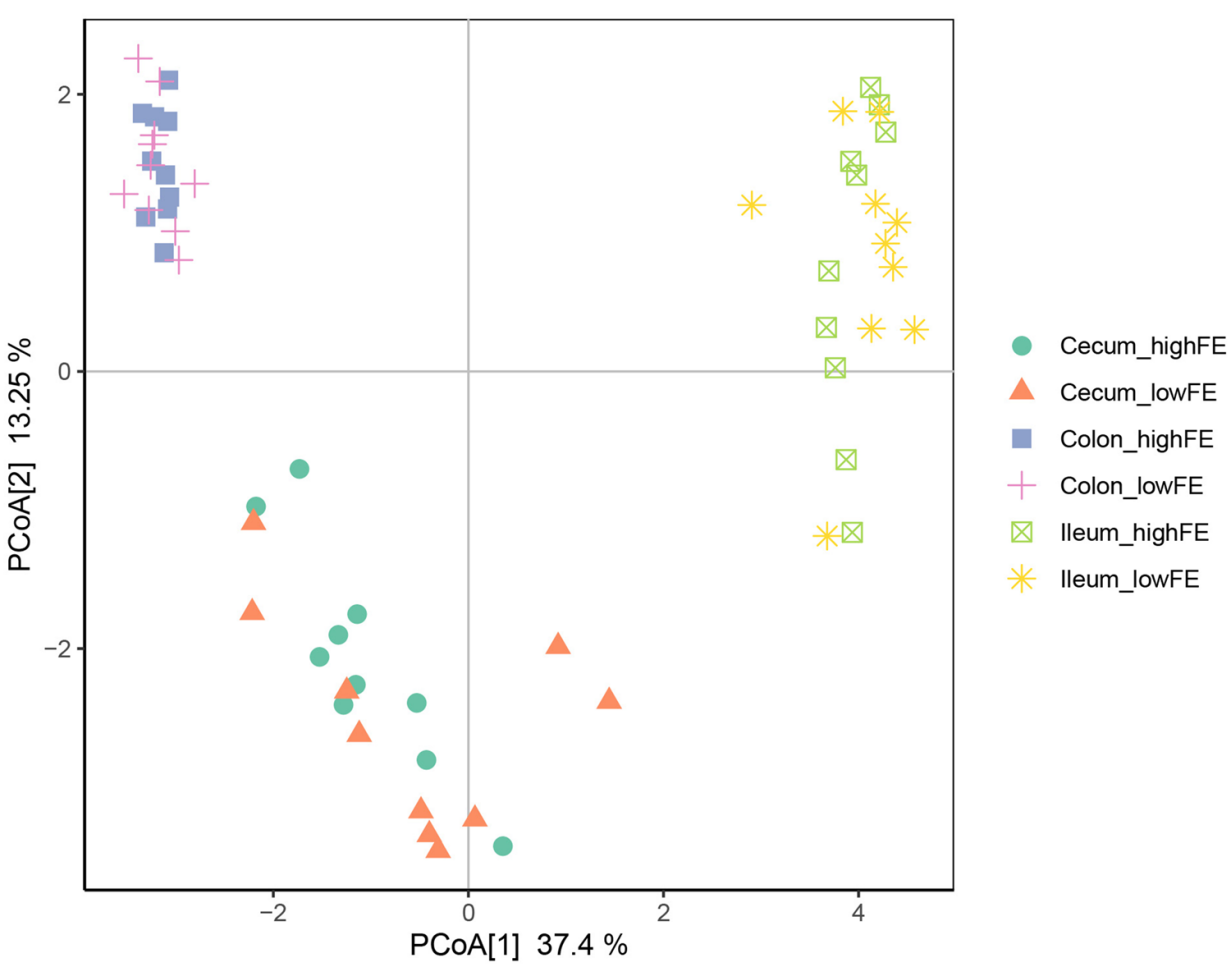
PCoA of the metabolites from distinct FCR groups.

To identify the small-molecule metabolites related to FE variation in pigs, we selected ten gilts with high FE and ten with low FE for metabolomic analysis of intestinal contents (Fig. 1). After quality control and annotation, a total of 749 metabolites were detected in samples from the three different intestinal locations and analyzed. Principal coordinate analysis (PCoA) analysis revealed strong separation of metabolites among the three locations, suggesting that the metabolic profile differs among these three intestinal segments. However, there was no clear separation of the samples between the high- and low-FE groups in any of the three locations (Fig. 2).

### Analysis of differential metabolites

**Figure 3:**
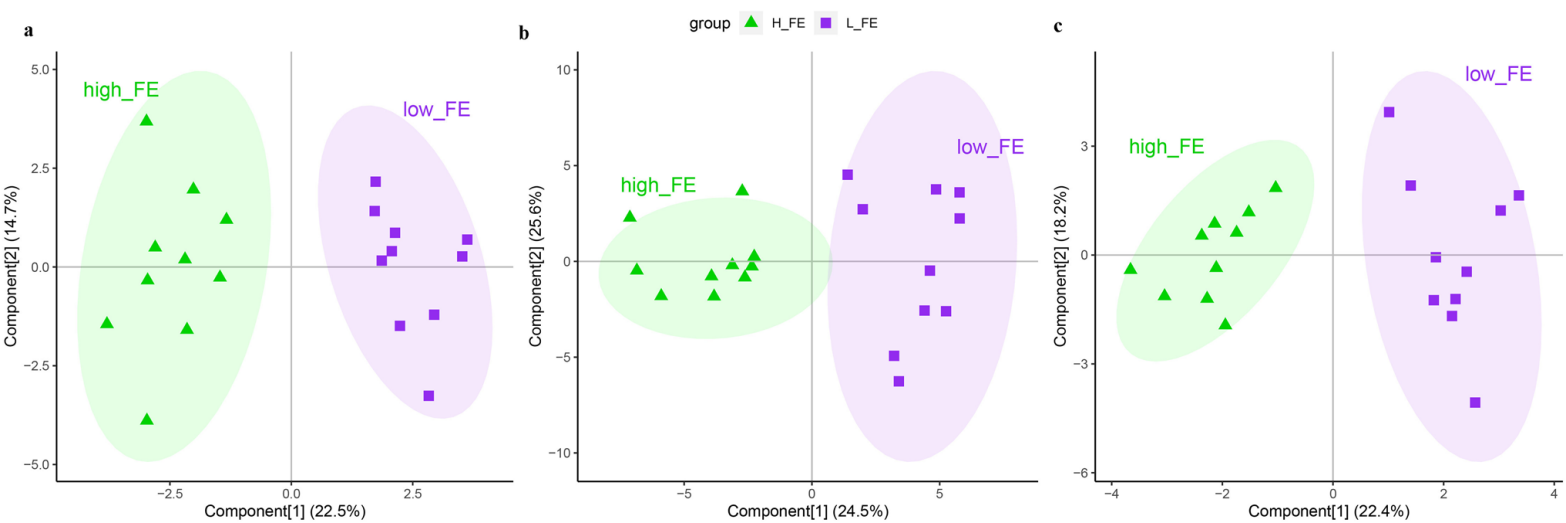
The OPLS-DA models discriminated between the HFE and LFE groups (R2Y of 0.93, 0.86 and 0.91 and Q2 of 0.73, 0.58 and 0.76 in ileum, cecum and colon, respectively). Permutation test (200 permutations) yielded pR2 < 0.05, pQ2 < 0.05 in each location (**a** ileum, **b** cecum, **c** colon).

**Figure 4:**
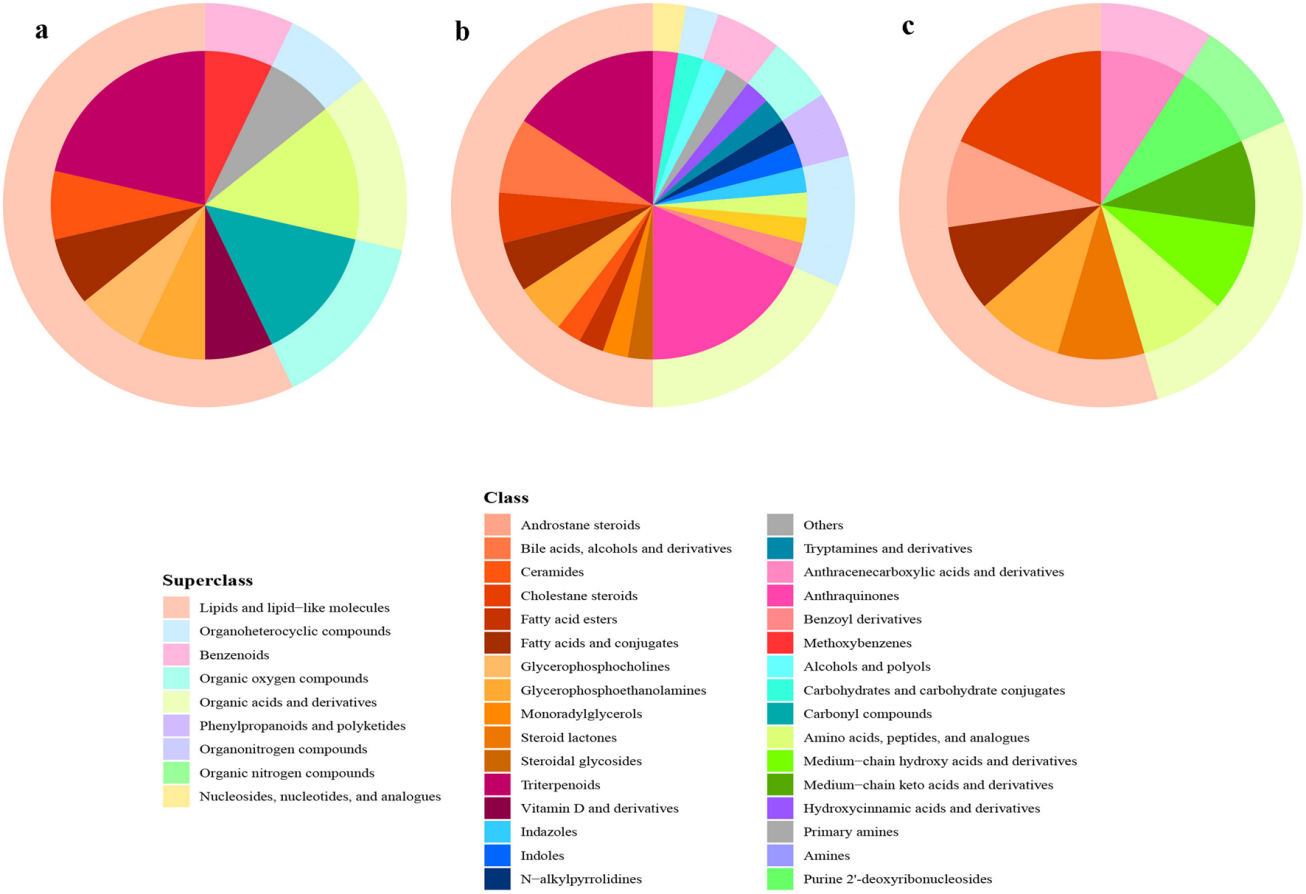
The biochemical categories of the differential metabolites identified between the high- and low-FE groups (**a** ileum, **b** cecum, **c** colon).

To identify the differential metabolites in each location between the two groups, initial screening of the metabolites was performed using ANOVA (*P* < 0.1)^13^. After the initial screening, 31, 84 and 25 metabolites in the three locations were selected. These metabolites were further screened according to VIP value from the OPLS-DA model (VIP > 1), yielding 15, 38 and 11 differential metabolites between the two groups for the three intestine locations (Fig. 3). These metabolites were classified into 9 major categories and more than 30 subcategories (Fig. 4).

### Metabolic pathway analysis

Metabolic pathway analysis of all the differential metabolites in each intestine location was performed. In cecum, pathway analysis identified 3 pathways that were enriched (tryptophan metabolism, steroid biosynthesis and purine metabolism), but none were significantly enriched. There were no enriched pathways in the ileum and colon (see Supplementary Fig.S1).

### Lipids and organic acids

We focused on the major categories of metabolites that likely contribute to feed efficiency in pigs, such as lipids and organic acids (Table 2). Lipids were identified in all intestinal locations, with prenol lipids, steroids and steroid derivatives composing the majority. In ileum and cecum, prenol lipids occurred at higher concentrations in the high-FE group. Steroids and steroid derivatives were found at higher concentrations in cecum but lower concentrations in colon in the high-FE group than in the low-FE group.

**Table 2:** Statistics of differentially accumulated lipids and organic acids in the high- and low-FE groups.

Class	Metabolites	VIP	M/Z	RT	Mode	*P* value	HFE/LFE
**Ileum**							
Lipids and lipid-like molecules	PC(22:5(4Z,7Z,10Z,13Z,16Z)/18:1(11Z))	1.134	834.6069	5.40	pos	0.079	Up
	Liquoric acid	1.344	484.3189	6.02	pos	0.023	Up
	Soyasapogenol C	1.034	441.3721	8.60	pos	0.464	Up
	Ganoderenic acid C	1.021	549.3439	4.30	pos	0.053	Up
	Cer(d18:0/16:0)	1.007	540.5352	11.44	pos	0.067	Up
	(23S)-23,25-dihdroxy-24-oxovitamine D3 23-(beta-glucuronide)	1.202	571.3255	4.29	pos	0.013	Up
	2-Octenoic acid	1.255	283.1911	7.19	neg	0.027	Down
	LysoPE(0:0/24:6(6Z,9Z,12Z,15Z,18Z,21Z))	1.094	576.3056	12.55	pos	0.033	Down
	12-Ketoporrigenin	1.428	467.2749	3.94	neg	0.031	Down
Organic acids and derivatives	Ceanothine E	1.078	613.3024	3.96	neg	0.066	Up
	5-octenoylglycine	1.138	232.1553	12.58	pos	0.017	Down
**Cecum**							
Lipids and lipid-like molecules	Cervonyl carnitine	1.037	472.3433	7.04	pos	0.062	Up
	FAHFA(18:0/9-O-18:0)	1.023	587.5005	11.07	neg	0.233	Up
	Dodecanedioic acid	1.109	483.2946	5.19	pos	0.108	Up
	MG(20:5(5Z,8Z,11Z,14Z,17Z)/0:0/0:0)	1.246	377.2687	5.46	pos	0.003	Up
	PE(18:4(6Z,9Z,12Z,15Z)/P-16:0)	1.168	678.4793	11.76	pos	0.013	Up
	PE(22:5(7Z,10Z,13Z,16Z,19Z)/P-18:0)	1.006	742.5563	9.68	pos	0.101	Up
	Ginsenoside Rf	1.316	835.4670	9.62	neg	0.047	Up
	Corosin	1.071	499.3094	7.31	neg	0.023	Up
	Isothankunic acid	1.052	503.3357	6.87	neg	0.138	Up
	3b,18b-3-Methoxy-11-oxo-12-oleanen-30-oic acid	1.037	449.3432	6.77	pos	0.039	Up
	(3beta,17alpha,23R)-17,23-Epoxy-3,29-dihydroxy-27-norlanost-8-ene-15,24-dione	1.032	473.3271	5.63	pos	0.024	Up
	Melilotoside A	1.012	555.4047	8.64	pos	0.872	Up
	Cer(d18:0/14:0)	1.079	512.5041	10.90	pos	0.302	Up
	Nutriacholic acid	1.172	355.2644	6.74	pos	0.422	Up
	3alpha,7alpha,12alpha,25-Tetrahydroxy-5beta-cholestane-24-one	1.155	492.3651	5.94	pos	0.034	Up
	Polyporusterone F	1.102	463.3456	6.69	pos	0.142	Up
	7-dehydrocholesterol	1.225	385.3575	10.47	pos	0.006	Up
	22,23-Dihydroergosterol	1.173	399.3631	10.65	pos	0.021	Up
	Aginoside progenin	1.216	805.4525	9.62	pos	0.162	Up
Organic acids and derivatives	Indicaxanthin	1.226	353.1004	4.33	neg	0.017	Up
	2-octenoylglycine	1.195	232.1553	13.45	pos	0.011	Up
	D-Pipecolic acid	1.192	130.0868	0.59	pos	0.063	Up
	5-Aminopentanoic acid	1.188	118.0867	0.87	pos	0.139	Up
	D-Lysine	1.106	147.1136	0.59	pos	0.126	Up
	5-octenoylglycine	1.138	232.1553	12.58	pos	0.023	Up
	Tryptophyl-Threonine	1.019	633.2607	3.01	pos	0.084	Up
**Colon**							
Lipids and lipid-like molecules	2-Octenoic acid	1.255	283.1911	7.19	neg	0.052	Up
	16-Oxoandrostenediol	1.196	305.2119	7.77	pos	0.108	Up
	LysoPE(18:0/0:0)	1.019	480.3089	8.97	neg	0.073	Down
	4,4-Dimethylcholesta-8,14,24-trienol	1.098	411.3627	8.12	pos	0.029	Down
	5a-Cholestane-3a,7a,12a,25-tetrol	1.058	459.3409	11.20	pos	0.032	Down
	2-Deoxybrassinolide	1.279	463.3410	8.74	neg	0.009	Down
Organic acids and derivatives	L-2-Amino-3-(1-pyrazolyl)propanoic acid	1.178	154.0620	0.64	neg	0.024	Up
	(S)-9-Hydroxy-10-undecenoic acid	1.140	245.1391	4.39	neg	0.053	Up
	3-Oxodecanoic acid	1.080	231.1236	3.86	neg	0.042	Up

*M/Z* mass-to-charge ratio, *RT* retention time, *VIP* variable importance in projection value.

The organic acids mainly comprised amino acids, peptides, and analogues in the ileum and cecum. These metabolites tended to occur at higher concentrations in the high-FE group. Three organic acids in colon occurred at higher levels in the high-FE group: a carboxylic acid, a hydroxy acid and a keto acid.

## Discussion

Global profiling of intestinal contents can provide insight into metabolic factors associated with feed efficiency^6,14^. Our study performed nontargeted metabolomics on content samples from three intestinal locations of pigs grouped into two feed-efficiency groups to identify potential FE-related intestinal metabolites. The metabolic profiles significantly differed among the different parts of the intestine. Metabolomics testing using the contents of a single part of the intestine cannot comprehensively reveal the host's intestinal metabolic activity. We identified a total of 785 metabolites in the three intestinal locations, of which 31, 84 and 25 were differentially accumulated in ileum, cecum and colon, respectively, between the two groups. As potential biomarkers of interest, we focused on lipids and organic acids, which have been considered to be related to FE in pigs in previous studies^15–17^.

The lipids identified in ileum and cecum included a variety of triterpenoid metabolites that occurred at higher concentrations in the high-FE group (liquoric acid, soyasapogenol C, and ganoderenic acid C in ileum and ginsenoside Rf, isothankunic acid, melilotoside A, and methyl glycyrrhetate in cecum). Triterpenoids are important natural products of plant origin that exhibit a wide range of biological activities, such as protecting the integrity of the intestinal barrier, regulating intestinal microbiota and inhibiting inflammation in the gastrointestinal tract^18–21^. The high levels of these metabolites in the high-FE group may partially explain the difference in FE between the groups.

In addition, several bile acids and provitamin D were identified in the cecum. Bile acids facilitate the absorption and metabolism of dietary lipids and fat-soluble vitamins^22^. Vitamin D (VD), as a fat-soluble prohormone steroid, improves calcium absorption, mediates the immune response and tempers inflammation in the intestine^23^. Previous studies have found that oral probiotics can increase VD levels in plasma^24^. In our study, the high-FE pigs had higher levels of secondary bile acids (nutriacholic acid) and provitamin D (7-dehydrocholesterol and 22,23-dihydroergosterol), which suggests a potentially increased conversion rate of bile acids from the primary to the secondary form and potentially increased efficiency of VD metabolism and utilization in high-FE pigs.

The organic acids in cecum with higher levels in the high-FE pigs were mainly amino acids and their derivatives. Cecal bacteria can use these amino acids for the synthesis of microbial proteins^25^. Among these organic acids, molecules related to the central inhibitory gamma-aminobutyric acid (GABA) systems were of particular interest (D-lysine, D-pipecolic acid and 5-aminopentanoic acid). Previous studies have shown that GABA has physiological functions, such as promoting animal feed intake and combatting stress^20,26^. Pipecolic acid and 5-aminopentanoic acid can be produced endogenously and through intestinal bacterial catabolism of lysine. Pipecolic acid is considered a neurotransmitter and plays a role in the central inhibitory GABA systems^27^. 5-aminovalerate, as a methylene homologue of GABA, functions as a weak GABA agonist^28^. Thus, our results suggest that the observed differences in these amino acids between groups contributed to the group differences in feeding efficiency.

Hydroxy fatty acids and oxo fatty acids are intermediates in intestinal microbial fatty acid metabolism^29^. Their higher colon concentrations in the high-FE pigs suggest that fatty acid biosynthesis and metabolism in colon might affect the feed efficiency of pigs.

## Conclusion

The aim of this work was to detect a molecular signature of the feed efficiency of DLY commercial pigs by performing untargeted metabolomics. This work provides comprehensive information regarding the metabolites in pig intestines. The molecular signature reveals the lipids and organic acids in intestine as important metabolites for feed efficiency. The identified differential lipids are mainly involved in combatting inflammation and oxidation in the ileum and cecum and in bile acid metabolism and vitamin D absorption in the cecum. The differences in organic acids were observed mainly in the hindgut, which is involved the metabolism of amino acids and fatty acids.

## Methods

### Ethics statement

All experimental procedures followed the ARRIVE guidelines (http://www.nc3rs.org.uk/arrive-guidelines). The 225 female DLY pigs in this study were all provided by Guangdong Wen's Foodstuffs Group Co., Ltd. (Yunfu, China). The care and use of animals in this study were approved and conducted according to standards established by the Animal Care and Use Committee (ACUC) of the South China Agriculture University (SCAU) (Guangzhou, China) (approval number SCAU#0032). The intestinal contents were sampled at the same intestinal locations in each pig that had been selected for slaughter. Briefly, the luminal contents of the ileum and colon were collected separately from the middle section, and the cecum contents were collected at the distal end of the cecum. All samples were collected within 30 min after slaughter and immediately placed in liquid nitrogen. The samples were returned to the laboratory and stored at − 80 °C for subsequent analysis.

### Phenotypic data collection

The feed intake and weight gain of all experimental pigs were recorded by an Osborne Feed Intake Recording Equipment (FIRE) Pig Performance Testing System (Osborne, KS, United States) in this study. These data were recorded when pig body weight (BW) was between 30 and 100 kg. The FCR value of each pig was calculated after the measurements of feed intake and weight gain were completed. RFI value was calculated by the method reported by Cai et al.^30^, which considers the midtest metabolic BW (MBW), average daily gain (ADG) and back fat (BF). The MBW was calculated as [(BW at on-test + BW at off-test)/2]0.75. Then, the FE performance of 225 animals was ranked according to FCR value, and 50 pigs with extreme FE were assigned two groups (25 highest FCR and 25 lowest FCR) and 10 pigs were randomly selected from each group to be slaughtered. The specific phenotypes of these 20 pigs are given in the Supplementary Table S1. Subsequently, the contents at three gut locations (ileum, cecum and colon) were collected from each pig. All samples were immediately stored in liquid nitrogen, transferred to the laboratory and stored at − 80 °C until LC–MS analysis. The R base package was used to detect differences in phenotypic traits between the two groups. The Shapiro–Wilk test was performed to examine the normality of the data. The Student t-test was applied for intergroup comparisons of normally distributed variables, and the Wilcoxon test was used for nonparametric variables. The results were considered significant at *P*-value < 0.05.

### LC–MS analysis

A total of 50 mg of each intestinal content sample was accurately weighed, and the metabolites were extracted using a 400 µL methanol: water (4:1, v/v) solution. The mixture was allowed to settle at − 20 °C and treated by high throughput tissue crusher Wonbio-96c (Shanghai Wanbo Biotechnology Co., Ltd) at 50 Hz for 6 min, followed by vortexing for 30 s and ultrasound at 40 kHz for 30 min at 5 °C. The samples were placed at − 20 °C for 30 min to allow the proteins to precipitate. After centrifugation at 13,000*g* at 4 °C for 15 min, the supernatant was carefully transferred to sample vials with 20 μL of 2-chloro-l-phenylalanine (0.3 mg/mL) for LC–MS/MS analysis. The Ultra Performance Liquid Chromatography (UPLC) system was coupled to a quadrupole-time-of-flight mass spectrometer (Triple TOFTM 5600+, AB Sciex, USA) equipped with an electrospray ionization (ESI) source operating in positive and negative mode. To monitor the stability of the analysis, quality control was prepared by two different methods. One is to extract and mix the same volume of each sample, the other is to set internal standard (2-chloro-l-phenylalanine). In the process of instrument analysis, a mixed sample was inserted every 8–10 samples. A relative standard deviation (RSD) of Internal standard < 30%, which represents the stability and repeatability of the system.

### Data processing and metabolite annotation

Peak detection and alignment of the raw data were performed with Progenesis QI 2.3 (Nonlinear Dynamics, Waters, USA), which generated a data matrix that included retention time (RT), mass-to-charge ratio (m/z) and peak intensity. The features detected in at least 50% of the samples were retained. After filtering out the low-coverage features, missing value was imputed according to the value in other samples by using the k-nearest neighbor (KNN) approach with the R DMwR package. Metabolic features with a relative standard deviation (RSD) of QC > 30% were discarded. The metabolic features value were normalized with a log10 transformation to better approximate a normal distribution. Mass spectra of the metabolic features were identified using the accurate mass, MS/MS fragment spectra and isotope ratio difference with searching in reliable biochemical databases such as the Human metabolome database (HMDB) (http://www.hmdb.ca/)^31^. Concretely, the mass tolerance between the measured m/z values and the exact mass of the components of interest was ± 10 ppm. For metabolites having MS/MS confirmation, only the ones with MS/MS fragments score above 30 were considered as confidently identified. Metabolome analysis data have been deposited to the Metabolights public repository under accession number MTBLS2512.

### Statistical analysis

Principal coordinate analysis (PCoA) was carried out using the vegan package of R. For the initial screening of features, an analysis of variance (ANOVA) was performed using the R base package. The metabolites that were significantly different between the two groups were selected (*P* < 0.1). Then, we screened these metabolites according to a threshold variable importance in projection (VIP) value (VIP > 1) from the orthogonal partial least-square discriminant analysis (OPLS-DA) model. OPLS-DA was performed using the R package ropls^32^. The OPLS-DA model quality was assessed using goodness of fit (R2) and goodness of prediction (Q2) in cross-validation via a permutation test with 200 permutations. Student's t-test was performed to assess the significance of differences in the abundance of metabolites (*P* = 0.05). Metabolite pathway analysis (MetPA) of all metabolites selected for each intestine location was performed with MetaboAnalyst 4.0 (www.metaboanalyst.ca)^33^. The metabolic pathways with *P*-value < 0.05 were considered significantly enriched.

## Supplementary Information

Supplementary Information
