# Supplementary material for: Lipids and organic acids in three gut locations affect feed efficiency of commercial pigs as revealed by LC–MS-based metabolomics
Source: Sci Rep. 2021 Apr 8;11:7746. doi: 10.1038/s41598-021-87322-8 (PMC8032704; doi:10.1038/s41598-021-87322-8)
Supplement: Supplementary file 1 — Supplementary Information [file 41598_2021_87322_MOESM1_ESM.docx]

Lipids and organic acids in three gut locations affect feed efficiency of commercial pigs as revealed by LC-MS-based metabolomics

Yong Ye^1, 2, +^, Jie Wu^1, 2, +^, Jianping Quan^1, 2^, Rongrong Ding^1, 2, 3^, Ming Yang^3^, Xingwang Wang^1, 2^, Shenping Zhou^1, 2^, Zhanwei Zhuang^1, 2^, Sixiu Huang^1, 2^, Ting Gu^1, 2^, Lingjun Hong^1, 2^, Enqin Zheng^1, 2^, Zhenfang Wu*^, 1, 2, 3^ and Jie Yang*^, 1, 2^

^+^ These authors contributed equally to this study

^1^ College of Animal Science and National Engineering Research Center for Breeding Swine Industry, South China Agricultural University, Guangdong, 510642, China.

^2^ Lingnan Guangdong Laboratory of Modern Agriculture, Guangzhou, 510642, China

^3^ Guangdong Wens Breeding Swine Technology Co., Ltd., Guangdong, 527400, China.

* Correspondence: Jie Yang: E-mail: [jieyang2012@hotmail.com](mailto:jieyang2012@hotmail.com); Zhenfang Wu: wzfemail@163.com

**Supporting Information**

**
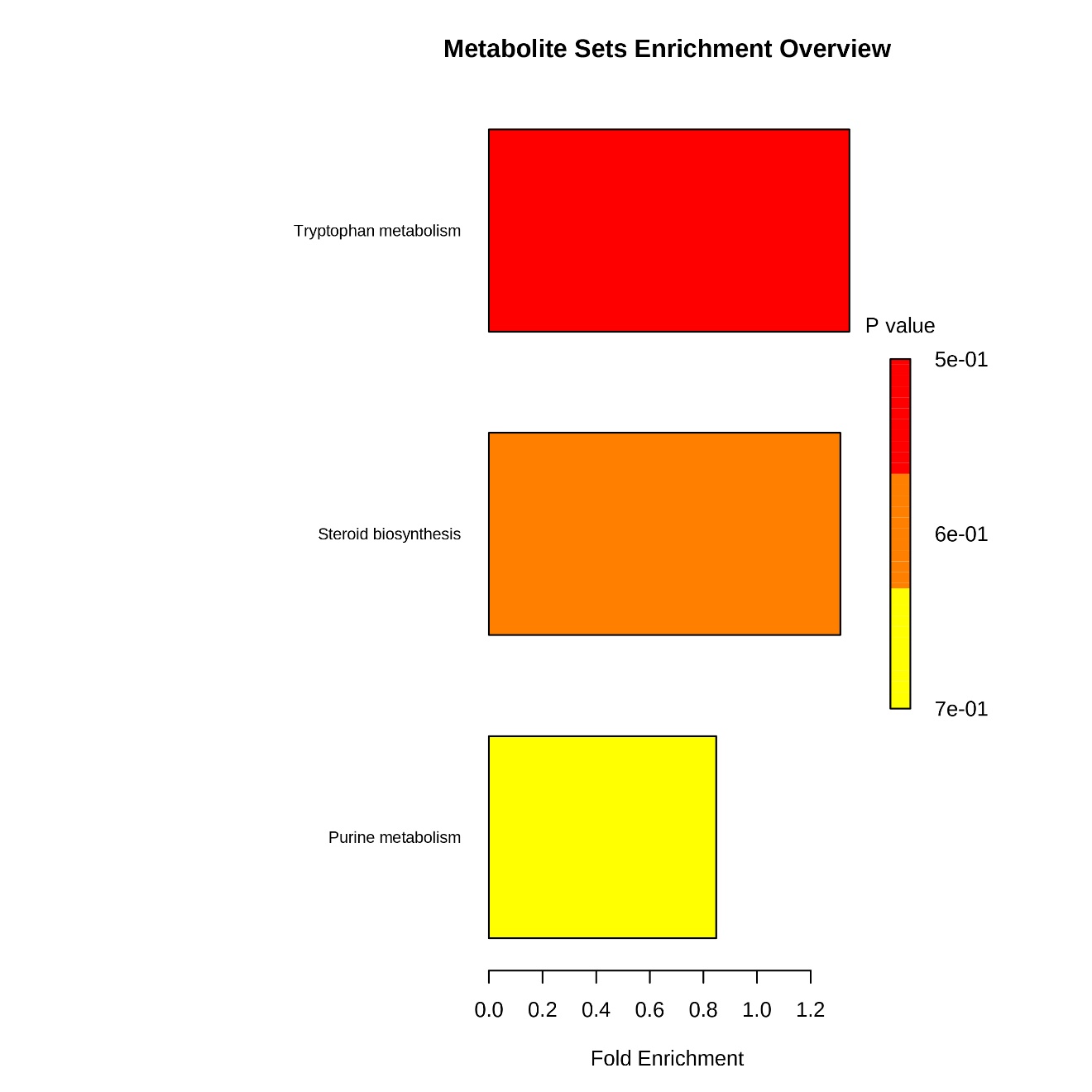
**

Figure S1 Path enrichment analysis of differential metabolites in cecum.

Table S1 Specific phenotype of 20 pigs with extreme feed efficiency.

| Sample.id | DFI | ADG | AMBW | RFI | FCR |
| --- | --- | --- | --- | --- | --- |
| HFE_1 | 1.779 | 0.824 | 23.129 | -0.203 | 2.185 |
| HFE_2 | 1.726 | 0.802 | 23.340 | -0.237 | 2.176 |
| HFE_3 | 2.063 | 0.932 | 23.327 | -0.082 | 2.245 |
| HFE_4 | 1.685 | 0.762 | 22.932 | -0.198 | 2.277 |
| HFE_5 | 1.975 | 0.921 | 22.813 | -0.128 | 2.280 |
| HFE_6 | 1.814 | 0.801 | 22.839 | -0.121 | 2.293 |
| HFE_7 | 1.675 | 0.824 | 22.786 | -0.289 | 2.051 |
| HFE_8 | 1.849 | 0.816 | 22.945 | -0.113 | 2.284 |
| HFE_9 | 1.938 | 0.872 | 23.011 | -0.106 | 2.262 |
| HFE_10 | 1.794 | 0.807 | 22.892 | -0.151 | 2.243 |
| LFE_1 | 2.222 | 0.880 | 22.998 | 0.168 | 2.618 |
| LFE_2 | 2.053 | 0.781 | 22.932 | 0.141 | 2.700 |
| LFE_3 | 2.278 | 0.828 | 22.918 | 0.301 | 2.775 |
| LFE_4 | 2.047 | 0.807 | 22.813 | 0.105 | 2.595 |
| LFE_5 | 1.979 | 0.766 | 22.720 | 0.101 | 2.606 |
| LFE_6 | 2.027 | 1.082 | 23.050 | -0.316 | 2.564 |
| LFE_7 | 1.839 | 0.757 | 22.667 | -0.024 | 2.593 |
| LFE_8 | 1.899 | 0.730 | 22.892 | 0.062 | 2.642 |
| LFE_9 | 1.930 | 0.743 | 23.077 | 0.064 | 2.652 |
| LFE_10 | 1.996 | 0.759 | 22.574 | 0.135 | 2.669 |

ADG, average daily gain; AMBW, average metabolic body weight gain; DFI, daily feed intake; RFI, residual feed intake; FCR, feed conversion ratio.
